# Characteristics of Recombinant *Chlamydomonas reinhardtii* Expressing Putative Germin-Like Protein from *Neopyropia yezoensis*

**DOI:** 10.4014/jmb.2407.07059

**Published:** 2024-08-14

**Authors:** Jiae Kim, Jong-il Choi

**Affiliations:** Department of Biotechnology and Bioengineering, Chonnam National University, Gwangju 61186, Republic of Korea

**Keywords:** Germin-like protein, *Chlamydomonas reinhardtii*, lipid, abiotic stress

## Abstract

Since microalgae face various environmental stresses for the high production of biofuels, multiple studies have been performed to determine if microalgae are resistant to these various stresses. In this study, the viability of cells under various abiotic stresses was investigated by introducing a putative germin-like protein (GLP) from *Neopyropia yezoensis*, which was known to be related in the resistance to abiotic stresses. The expression of GLP in *Chlamydomonas reinhardtii* allowed cells to grow better in various abiotic stress environments. In nitrogen starvation conditions, recombinant cells accumulated the lipid droplet 1.46-fold more than wild-type cells and responded more rapidly to form palmelloid forms. Under high-temperature, hydrogen peroxide conditions and saline stress, the survival rate was increased 3.5 times, 2.19 times, and 3.19 times in recombinant *C. reinhardtii* with GLP, respectively. The expression level of genes related to pathways in response to various stresses increased 2-fold more under those conditions. This result will be useful for the development of microalgae that can grow better and produce more biofuels under different stress conditions.

## Introduction

Widespread increasing temperature of 1.5 – 2°C and reduced precipitation are anticipated by the end of the 21st century [1], resulting in decreased runoff and increased salinization of epicontinental for freshwater bodies. Since the increased potential of salinity to alter the structure of phytoplankton communities, the short-term and rapid response of freshwater phytoplankton to various stresses has been extensively addressed [2]. *C. reinhardtii* is a popular model organism for phytoplankton with a fully sequenced and well-annotated genome; meanwhile, various tools exist to modify and characterize their genome [3, 4]. *C. reinhardtii*, a single-celled eukaryotic microalga inhabiting both soil and aquatic environments, relies on CO_2_ and light for growth, thriving within the mesophilic temperature range of 20 – 32°C. Extended exposure to incompatible temperatures leads to metabolic cessation, chlorosis, and, ultimately, cell death [5].

Similar to these studies, when microalgae undergo abiotic stress such as nutrient deprivation, high temperature, and salinity stress, they accumulate large quantities of lipids for survival [6]. In moderate stress conditions, acclimation of microalgae is induced. Exposure to harsher stresses will enable *Chlamydomonas* to activate a range of alternate strategies involving interaction between multiple cells and/or programmed cell death. Furthermore, *Chlamydomonas* can form multicellular structures such as palmelloids or aggregates that can dissociate when environmental conditions improve [7]. Under moderate stress conditions, where the cell can survive, microalgae accumulate lipids that can potentially act as third-generation biomass to be the next energy source. The lipids that microorganisms accumulate within their cells are used to produce a variety of biofuels, such as biodiesel and ethanol. Microalgae offer numerous advantages over traditional first- and second-generation biomass in that they require less water than conventional crops, thrive in saline wastewater environments, and do not cause issues related to arable land scarcity [8]. These attributes make microalgae cultivation highly attractive for sustainable biomass production and biofuel generation. However, there is a problem with cell growth being inhibited. Eventually, reduced cell growth will lead to lower lipid productivity, even if the lipid content is enhanced [9]. Therefore, efforts to identify different microalgae strategies for resisting and surviving multiple abiotic stresses, such as obtaining creating by introducing various genes resistant to abiotic stress, have been extensively studied [10-13].

In this study, we characterized the gene of putative germin-like protein (GLP) found in *Neopyropia yezoensis* and investigated the effect by introducing the gene into *C. reinhardtii*. Resistance tests to various abiotic stresses were performed using the transformed *C. reinhardtii*, and quantitative experiments were performed on numerous responses to abiotic stress using qRT-PCR.

## Materials and Methods

### Strains and Growth Condition

This study used *Escherichia coli* DH10B (Invitrogen, USA) as the gene cloning host. Then, we used wild-type *C. reinhardtii* cc-124 to compare lipid accumulation and growth under various abiotic stress conditions. *E. coli* DH10B were cultured in Luria–Bertani (10 g/l yeast extract, 5 g/l tryptone, and 10 g/l NaCl) medium with ampicillin of 100 μg/ml. *C. reinhardtii* cells were incubated in Tris-acetate-phosphate (TAP) medium containing 2.42 g Tris-base, 25 ml TAP-salts, 0.375 ml phosphate solution, 1 ml Hutner’s trace elements solution, and 1 ml acetic acid per L. The detailed contents for the solution is described in [11]. The NH_4_Cl in TAP salts was replaced with KCl (TAP-N) for the nitrogen-derived medium. The cells were cultured at 25°C with shaking at 150 rpm under continuous cool white light (40 μmol protons m^-2^s^-1^) while nitrogen starvation was performed under 12:12 h (L:D) conditions.

### Multiple Sequence Alignment

A previous study annotated a gene as GLP from *N. yezoensis* (unpublished data). In this study, we characterized this gene by comparing the GLP sequence with those of other species using Clustal Omega Multiple Sequence Alignment (MSA) (version 1.2.4).

### Construction of Vector and Recombinant *C. reinhardtii* with GLP

The target gene was cloned into the pCr102 vector, which was used as an expression vector in *Chlamydomonas*. The GLP and vector sequences were used to design specific primers for cloning using the Gibson assembly method (Table 1). The recombinant plasmid pCr102_GLP contained psaD promoter as a housekeeping promoter and terminator for GLP expression. The *β2-Tub* promoter was contained to express *Aph7"*, which is inactive hygromycin, meaning we can select the transformant. Then, we transformed this plasmid, resulting in ampicillin resistance in the *E. coli* DH10B strain, which can be used to determine that plasmid construction is complete. To transfer this plasmid into *C. reinhardtii*, cells were pre-cultured and main-cultured for 3 days each in TAP media to reach the mid-log phase (~1.0 × 10^6^ – 2.0 × 10^6^ cells/ml). Next, they were centrifugated at 2,500 rpm for 5 min and then washed with 10 ml of MAX Efficiency™ Transformation Reagent for Algae (Invitrogen). Each cell was divided into 250 μl, and 2 – 4 μg of DNA was mixed in a Gene Pulser Cuvette (Bio-Rad, USA). Electroporation was executed at 500 V voltage, 50 μF capacity, and 800 Ω resistance. After electroporation, the transformed cells were incubated at room temperature for 1 min and recovered with TAP (40 mM) sucrose solution for 14 – 16 h while constantly shaking at 150 rpm under white light. After the recovery stage at room temperature, the cells were centrifugated at 2,500 rpm for 5 min; the pellet was suspended in 200 μl TAP medium and plated on TAP agar medium containing hygromycin B (50 μg/ml) for 3 days.

### Quantitative Real-time PCR

To confirm if the cloned plasmid was transformed into the cells, quantitative real-time PCR (qRT-PCR) was performed. The recombinant cell was pre-cultured for 3 days and centrifuged at 2,500 rpm for 5 min. After clearly removing the supernatant, cells were resuspended with 1 ml Trizol^®^ LS Reagent (Ambion Inc., USA) and homogenized for 10 min by vortexing. Then, they centrifugated at 12,000 ×*g* for 10 min at 4°C, and the supernatant was mixed with 200 μl of chloroform, vortexed for 2 min, and centrifuged again. The aqueous phase containing the RNA was transferred to a new tube, and 0.5 ml of isopropanol was added. Then, the tube was incubated on ice for 1 h. The mixture was centrifuged at 7,500 ×*g* for 20 min; then, the precipitated RNA pellet was washed with 75% ethanol (v/v) and fabricated with diethyl pyrocarbonate water (DEPC-DW). The pellet was dried at room temperature for 5 – 7 min and added to 50 μl of DEPC-DW. The pellet was resuspended naturally when reacted at 65°C for 5 – 10 min. cDNA was synthesized using a first-strand cDNA synthesis kit. The transcription level was analyzed using TB green premix Ex Taq (Takara Bio Inc., Japan) following the manufacturer’s instructions. A Real-Time PCR System (Illumina Inc., USA) was used to observe the expression change. The used primers are described in Table 1 [9].

### Measurement of Lipid Droplet and mRNA levels

The nitrogen starvation stress was administered to analyze the effect of the GLP gene in *C. reinhardtii* in response to nitrogen stress. The *C. reinhardtii* wild-type control (Cr_control) and recombinant *C. reinhardtii* transformed with GLP (Cr_GLP) were pre-cultured in 10 ml TAP broth. A total of 1 ml of seed culture was inoculated in 100 ml of TAP medium (with N+) and cultured for 3 days. After the main culture, the 100 ml of cell culture was divided into the same volume and washed using N+ medium and N– medium. Then, the cell cultures were inoculated into 100 ml TAP N+ and TAP N– medium and cultured for 7 days.

Depending on the incubation time, cells from days 0, 3, and 7 were sampled and used for lipid droplet and starch observation. Lipid production in the strains was confirmed qualitatively using microscopy and quantified following previous protocols [9]. The 375 μl of cell culture was mixed with 125 μl dimethyl sulfoxide (DMSO), and the 2 μl Nile Red solution was used to stain the cells. The Nile Red solution was made with 2.5 mg Nile Red (Sigma-Aldrich, USA) in 10 ml DMSO. After incubation for 10 min, the cells were centrifuged at 400 ×*g* for 1 min and washed using D.W. Stained samples were observed using a phase contrast microscope with a fluorescent lamp. Then, relative quantification of mRNA levels related to starch degradation and lipid accumulation was conducted. The primers used in qRT-PCR are described in Table 1.

### Cell Growth and Survival Rates under Abiotic Stress

The *C. reinhardtii* wild-type control (Cr_control) and recombinant *C. reinhardtii* transformed with GLP (Cr_GLP) were pre-cultured in 10 ml TAP broth. The pre-cultured medium was washed using phosphate-buffered saline solution (PBS, 1×). To measure the tolerance of Cr_control and Cr_GLP, cells were diluted up to 10^-7^ and spotted onto TAP agar plates at 5 μl each. For the hydrogen-peroxide (H_2_O_2_) or saline-stress conditions, H_2_O_2_ or sodium chloride (NaCl) was added to the TAP-agar medium with accurate concentrations. The H_2_O_2_ solution was sterilized using a PTFE membrane with 0.22 μm pores before being added to the medium. The growth check following H_2_O_2_ stress was performed with concentrations of 0, 0.2, 0.4, and 0.6 mM, and NaCl was added at 0, 100, 200, and 300 mM. Survival rate in saline stress and H_2_O_2_ was calculated by comparing the number of colonies at 0 mM with the number of colonies under the corresponding conditions.

High-shock stress was performed at temperatures of 38°C, 40°C, and 42°C. The pre-cultured medium was incubated for 2 h at three temperatures, and sampling was conducted for comparison after 1 h. Thereafter, the dotting process was performed similarly to the one mentioned above. The cells were cultured at 25°C for 3 days under continuous cool white fluorescent light. In addition, the survival rate under heat stress conditions was calculated based on the number of colonies for 0 h of cultures sampled immediately after inoculation of the precultured culture at 25°C [13]. Previously reported studies were referred to design the experiment [13].

### Expression Levels of Various Genes Related to Stress Response

The Cr_GLP and Cr_control was pre-cultured for 3 days, and optical density was checked at 750 nm using a UV spectrophotometer. The optical density value was diluted to 0.1 and inoculated into various mediums.

A total culture volume of 10 ml was used, diluted to an optical density value of 0.1, and incubated under high-temperature stress conditions of 38°C for 1 h and 2 h. The recovery process was performed under 25°C and continuous cool white light for 12 h. A total of 1 ml of this dilution culture was inoculated to an optical density value of 0.1 and added into the 9 ml TAP medium with an accurate concentration of H_2_O_2_ and NaCl. The culture medium was prepared in the same manner as described above. Then, the culture was incubated at 25°C under continuous cool white light for 12 h. After the recovery and incubation, RNA preparation and cDNA synthesis were carried out to quantify the expression of genes related to the stress responses. The primers used are described in Table 1 [14].

### Statistical Analysis

All experiments were carried out in triplicate. Statistical analysis was performed using t-Student test (GraphPad Software, USA). Data is expressed as mean ± standard error of the mean (SEM). In all statistical analyses, an asterisk (*), two asterisks (**), and three asterisks (***) denote statistical significance at the *p* < 0.05, *p* < 0.01, and *p* < 0.001 levels, respectively.

## Results

### Multiple Sequence Alignment of GLP_Gene and Construction of Recombinant Strain

**Fig. 1:**
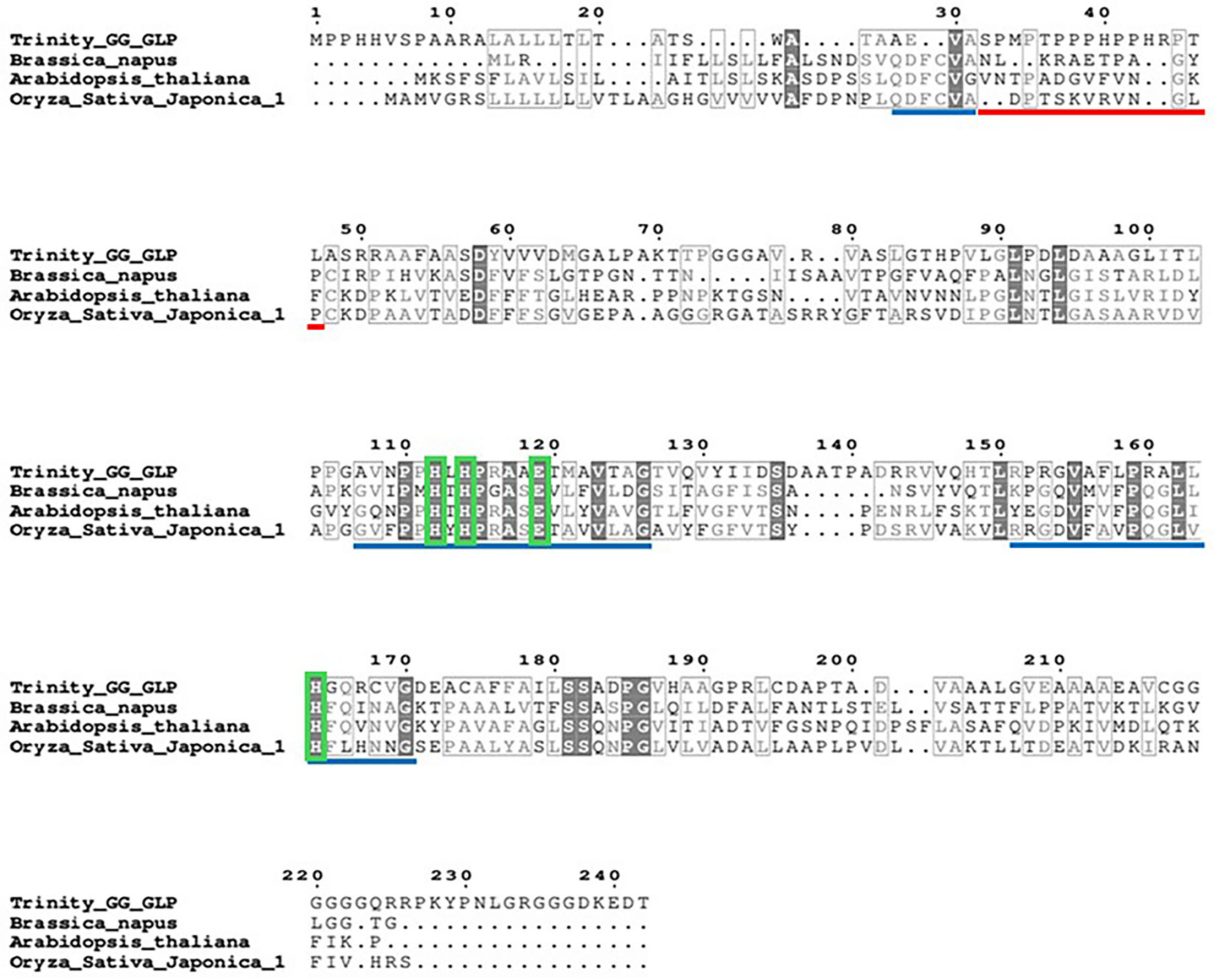
Multiple sequence alignment of GLP gene from the *N. yezoensis* Daebudo and other species with the highest identification percentage. These species were *B. napus* (P46271.1), *A. thaliana* (Q9FMB0.1), and *O. Sativa* (Q688L5.1) (P46271.1), respectively. The light blue line indicates the sequences having very high sequence conservation, and the sequences with the red line are highly variable regions. Light green indicates the amino acids involved in binding a metal ion.

**Fig. 2:**
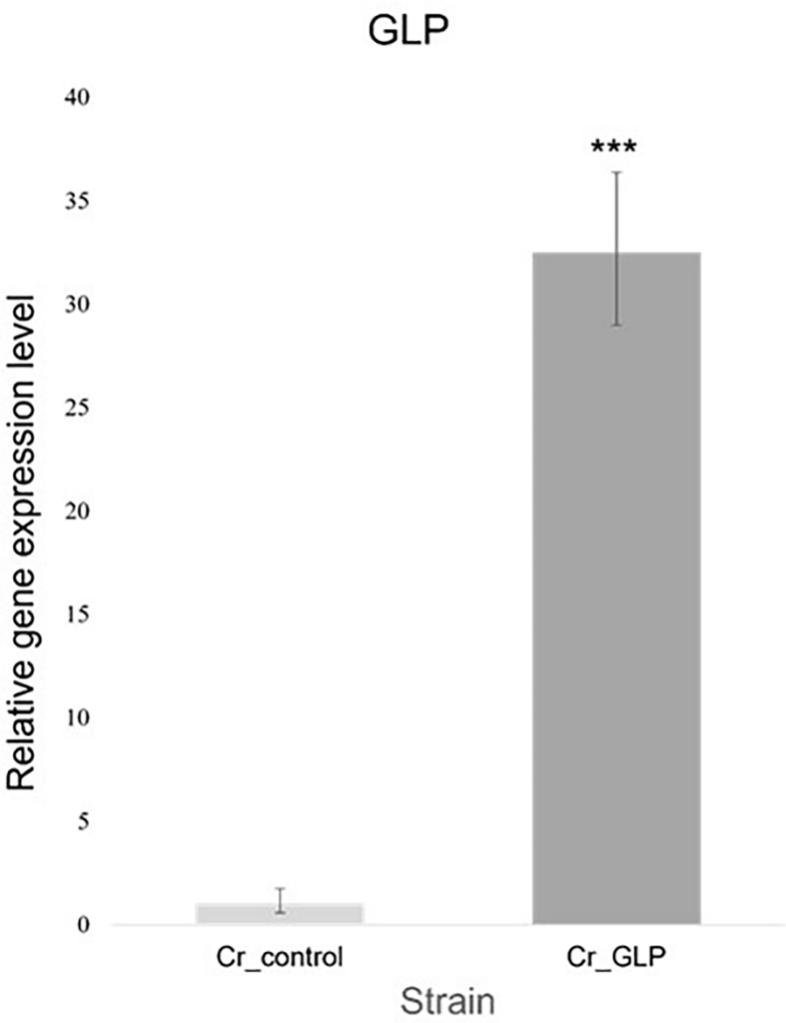
Relative expression levels of GLP in Cr_control (WT) and recombinant Cr_GLP, repectively.

Putative GLP gene was identified in the comparative transcriptome analysis of *N. yezoensis* Daebudo. In the newly isolated *N. yezoensis* species with high growth at high temperate, putative GLP gene was shown to be overexpressed (data not shown). We used BlastX with Swissprot_database results for the putative GLP gene from *N. yezoensis* to compare with those of other species. Conservation levels of 40.51%, 40.37%, and 37.21% were obtained for *Arabidopsis thaliana*, *Oryza Sativa*, and *Brassica napus*, respectively. Conserved regions and regions that affect the activation of GLP are indicated in Fig. 1. The *C. reinhardtii* CC-124 wild-type strain was used to be transformed with the constructed vector pCr102_GLP. Then, qRT-PCR was performed to choose a recombinant Cr_GLP (Fig. 2).

### Comparison of Growth and Lipid Accumulation under Nitrogen Deprivation Conditions

**Fig. 3:**
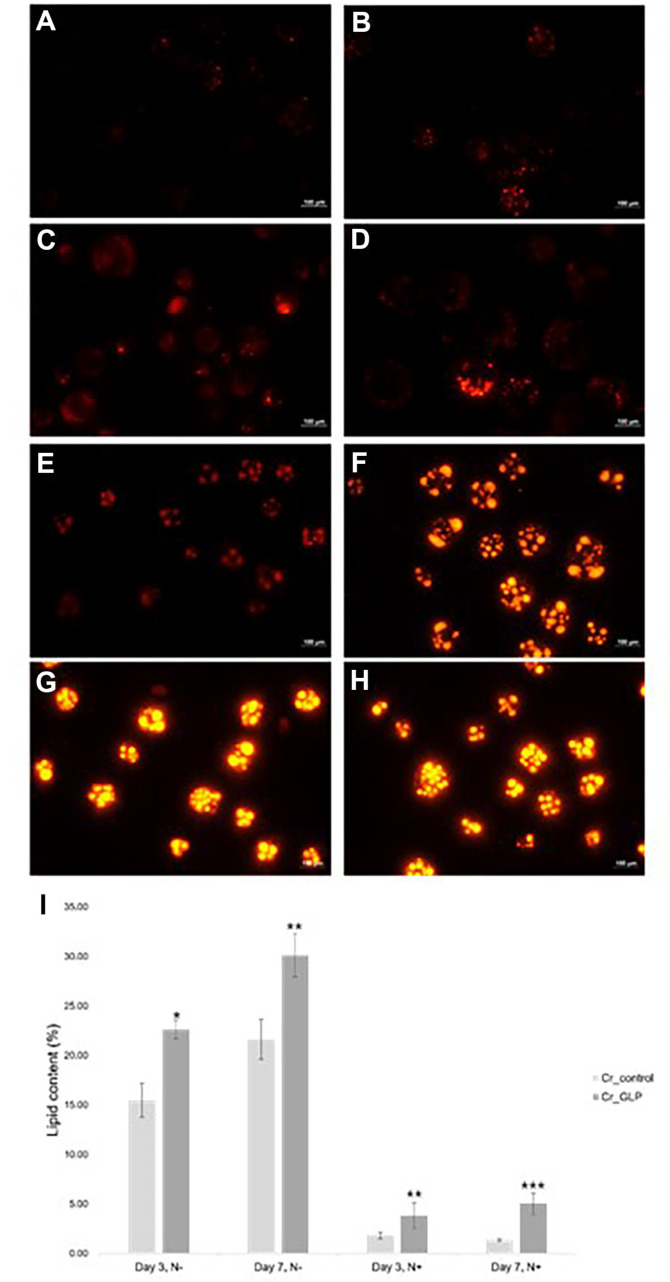
Microscopic images and quantitative results of lipid accumulation in Cr_control (A, C, E, G) and Cr_GLP (B, D, F, H) under normal and nitrogen starvation conditions, respectively. With nitrogen for 3 days (**A, B**), with nitrogen for 7 days (**C, D**), without nitrogen for 3 days (**E, F**), and without nitrogen for 7 days (**G, H**), respectively. The quantitative lipid contents were shown in Fig. 3 (**I**).

The growth and lipid accumulation of *C. reinhardtii* were analyzed under nitrogen starvation conditions to evaluate the effect of the GLP protein expression. Since *C. reinhardtii* accumulates lipids in the body under nitrogen-derived conditions, lipid accumulations were compared. The microscopic observation was shown in Fig. 3. Under nitrogen starvation conditions, Cr_GLP accumulated 22.63% of the lipids over 3 days and 30.14%over 7 days, whereas the Cr_control accumulated 15.48% and 21.64%, respectively. In normal conditions, the lipid accumulations of Cr_GLP were 1.78% and 1.79% over 3 and 7 days, respectively. Likewise, the lipid contents of the Cr_control were 2.06% and 1.39%, respectively (Fig. 3I). When cultured for 3 days in nitrogen starvation conditions, Cr_GLP accumulated 1.46-fold more lipids than the wild-type (Cr_control), while accumulation was 1.39 times higher than the Cr_control after 7 days. However, the difference in lipid content in the normal TAP medium was not significant owing to the increased effect of GLP after introduction into *C. reinhardtii* in stressful situations such as nitrogen deprivation than in normal conditions.

**Fig. 4:**
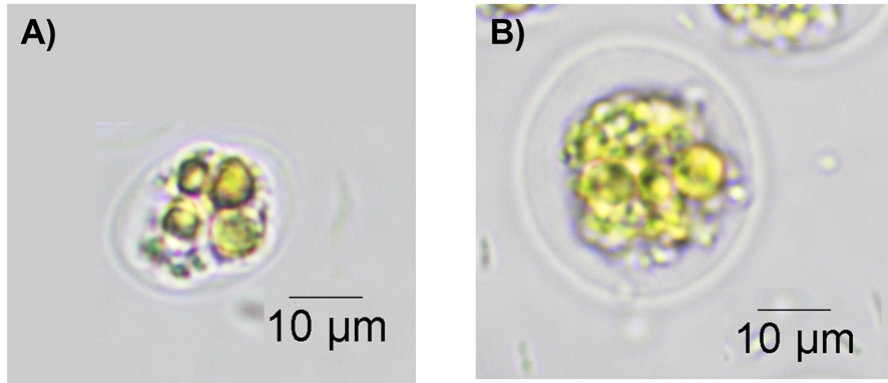
Microscopic observation of cells. The Cr_GLP (A) shows such a pallmeloid form faster than Cr_control (B) under 3 day-culture.

In response to nitrogen deprivation conditions, *C. reinhardtii* has a palmelloid form that does not divide and divides within the cell wall. As a result of microscopic observation of 3 days, the palmelloid form was more identified in the Cr_GLP strain than in the Cr_control strain. Through this, it may be seen that the strain introduced with GLP gene responded to the stress condition more rapidly (Fig. 4).

### mRNA Levels Related to Starch Degradation and Lipid Accumulation

**Fig. 5:**
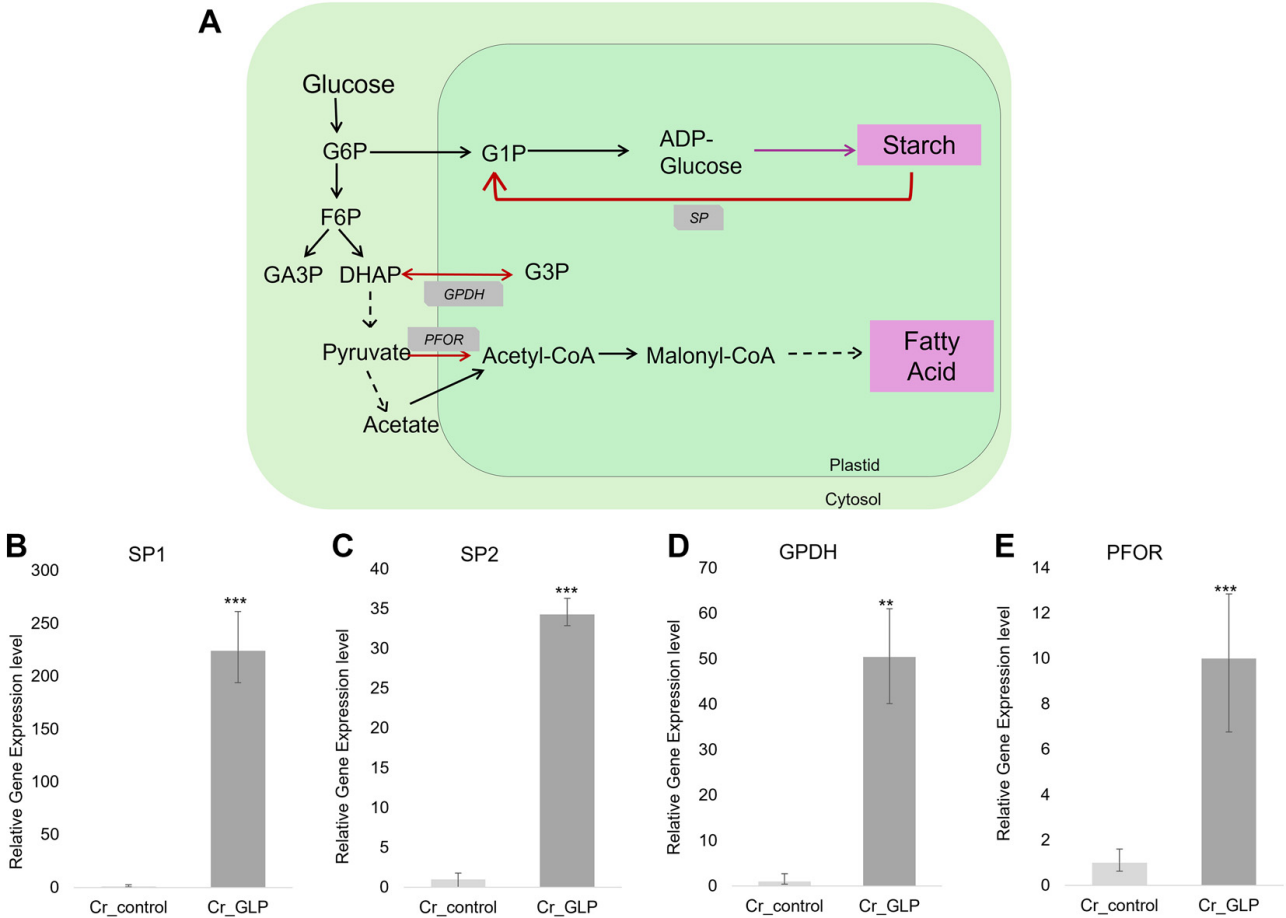
Schematic diagram for starch and lipid synthesis pathway (A) and relative gene expression levels for SP1 (B), SP2 (C), GPDH (D), and PFOR (E) of *Chlamydomonas* strains under nitrogen limitation condition, respectively.

Growth-arrested *Chlamydomonas* cells store a polysaccharide that closely resembles the storage starch found in higher plants, both structurally and functionally. It serves as a storage carbohydrate that *Chlamydomonas* uses during nutrient limitation or stress conditions, similar to how plants use starch for energy storage [43]. Moreover, microalgae accumulate energy-rich compounds such as lipids and starch during environmental stress, such as nitrogen deprivation, by redirecting carbon towards these compounds for storage [44-46]. Starch is synthesized for a short-term energy source, and then lipid is synthesized for a long-term energy storage compound [38]. However, previous studies have shown that the lipid content of microalgae can be increased by redirecting carbon partitioning from starch to lipid synthesis through the knockout of starch-related genes [47]. Carbon partitioning from CO_2_ to starch and lipid accumulation and the related key enzymes in *Chlamydomonas* are shown in Fig. 5A. In this study, lipid accumulation increased over time, and thus, the expression level for the key enzyme in mechanisms for lipids and starches was compared. In this study, the starch concentration could not be measured in *C. reinhardtii* cells because of low concentration and small volume. Therefore, to investigate the relationship between starch degradation and lipid accumulation, the gene expression levels were compared. Starch phosphorylase (SP), which is related to starch degradation mechanisms, glycerol-3-phosphate dehydrogenase (GPDH), which serves as a link between carbohydrate metabolism and lipid metabolism, and pyruvate, ferredoxin oxidoreductase (PFOR), which catalyzes the interconversion of pyruvate and acetyl-CoA is used as the key enzyme. SP expression in the Cr_GLP increased up to 220 times compared to the Cr_control, while GPDH and PFOR increased by about 50 and 10 times, respectively (Fig. 5).

### Comparison of the Cell Survival Rate under Abiotic Stress

**Fig. 6:**
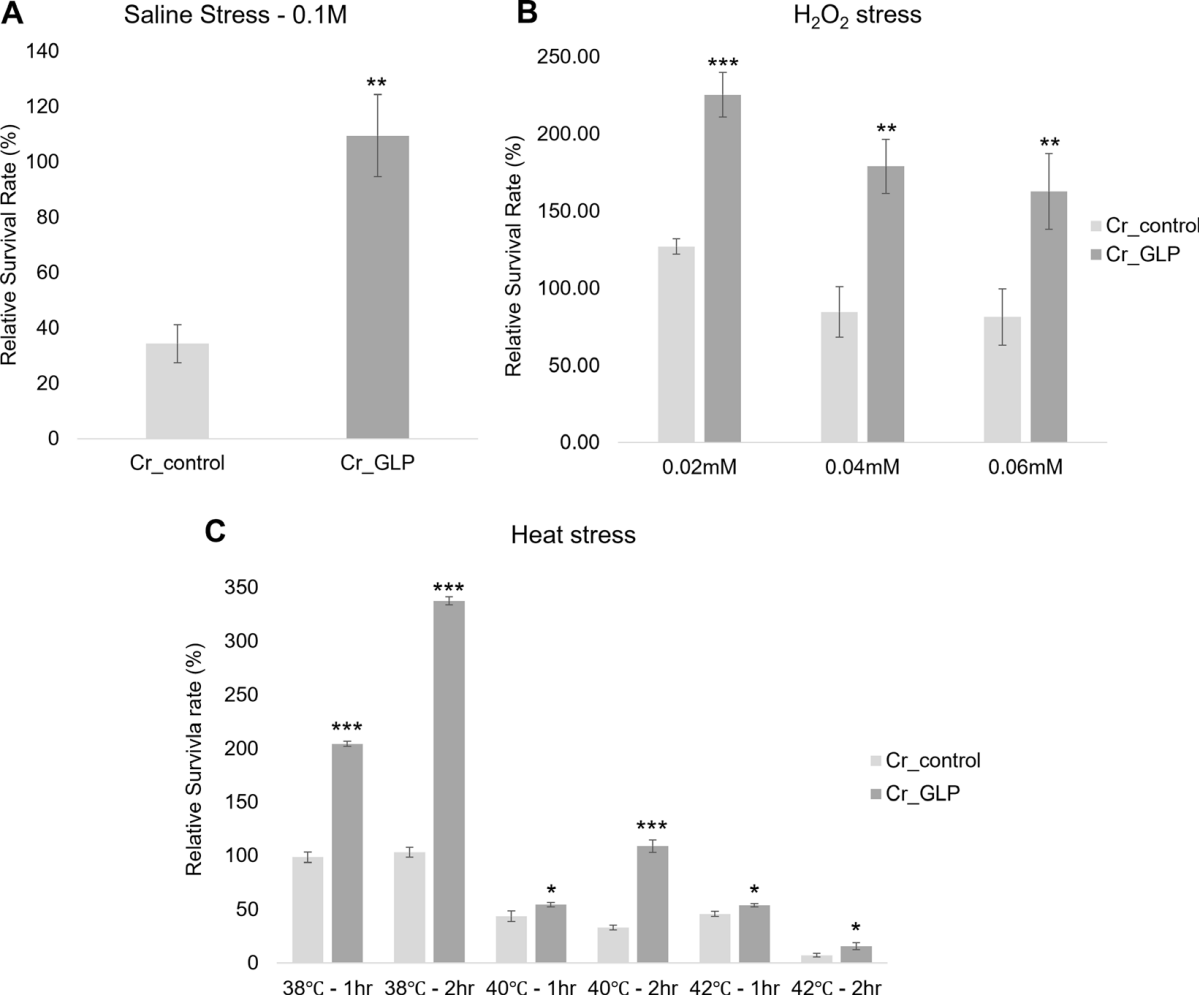
Survival rates of *Chlamydomonas* strains under saline stress (A) H_2_O_2_ stress (B) and heat stress (C) conditions, respectively.

Since GLP is known to resist various abiotic stresses, growth confirmation has progressed during various stresses. In the TAP agar medium containing 0.1 M NaCl, Cr_GLP had a 3 times higher survival rate with a ratio of 110% while the Cr_control was 34%. Neither the Cr_control nor the Cr_GLP grew in the TAP agar medium containing NaCl beyond that concentration (Fig. 6A).

In the survival rate experiment under H_2_O_2_ stress, Cr_GLP showed survival rates of 225.6%, 179.07%, and 162.79% for H_2_O_2_ at 0.02 mM, 0.04 mM, and 0.06 mM, respectively, while the Cr_control exhibited survival rates of 127.12%, 84.75%, and 81.36%, respectively, for the same concentrations. Cr_GLP survived better than Cr_control, which only survived at an H_2_O_2_ concentration of 0.02mM (Fig. 6B).

When both cells faced heat stress, Cr_GLP had an almost 3.5 times higher survival rate than Cr_control. When Cr_GLP was incubated at 38°C and 40°C for 2 h, the number of colonies increased compared to the control, which only grows at optimal conditions. As the temperature increased, the number of colonies decreased, yet the longer the exposure time to high temperatures, the higher the survival rate. Detailed data are shown in Fig. 6C.

### Expression Levels of Genes involved in Various Stress-Response Mechanisms

**Fig. 7:**
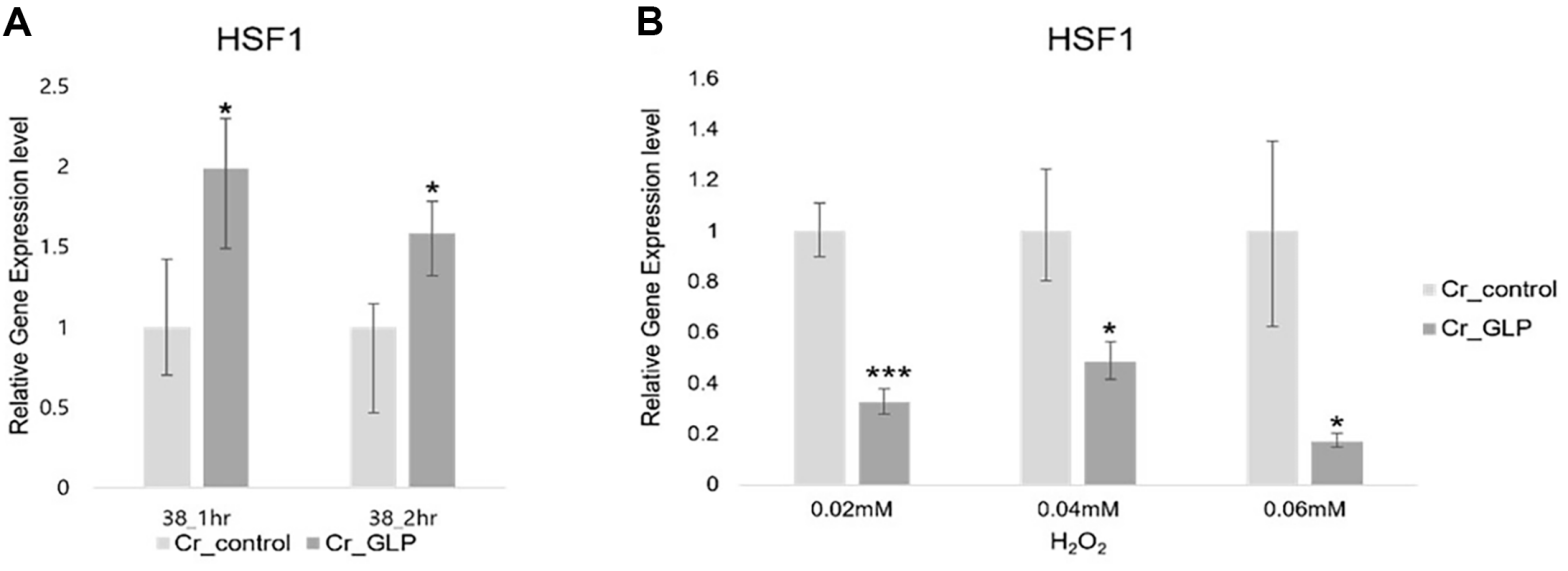
Relative gene expression level of HSF1 of *Chlamydomonas* strains under heat (A) and H_2_O_2_ stresses (B) conditions, respectively.

Quantitative measurements for stress-response genes were performed under various stress conditions. Since GLP is a protein introduced from *N. yezoensis* daebudo, which is resistant to high temperature, qPCR was performed on the heat shock transcription factor1 (HSF1), which stimulates the transcription of heat shock proteins [15, 16]. HSF1 was, on average, expressed in Cr_GLP up to 2-times more than the Cr_control (Fig. 7A), whereas it was expressed less than the wild-type in the H_2_O_2_ condition (Fig. 7B)

**Fig. 8:**
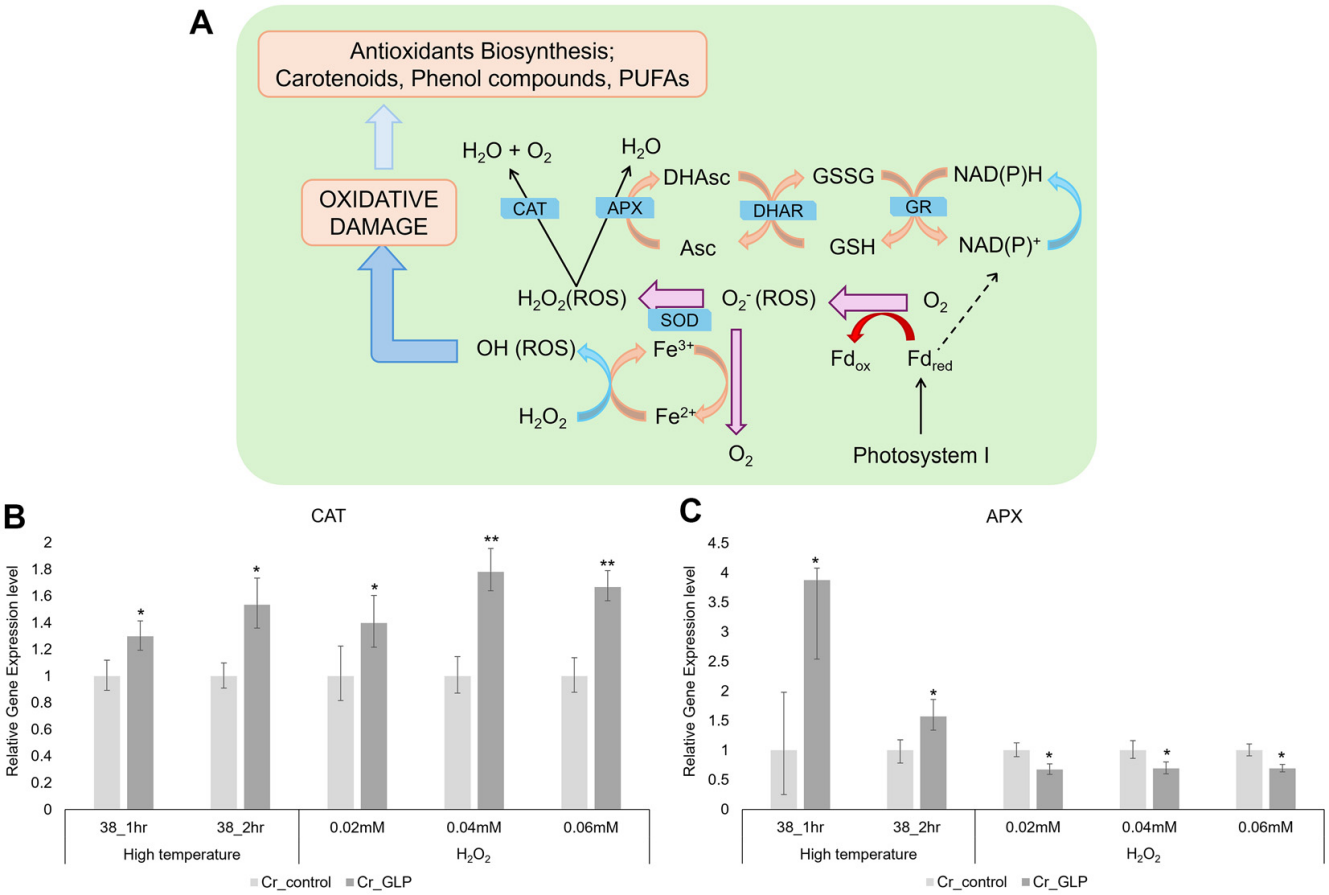
Schematic diagram of relate genes for ROS-scavenging pathway (A) and relative gene expression levels of CAT (B) and APX (C) under heat and H_2_O_2_ stresses conditions, respectively.

**Fig. 9:**
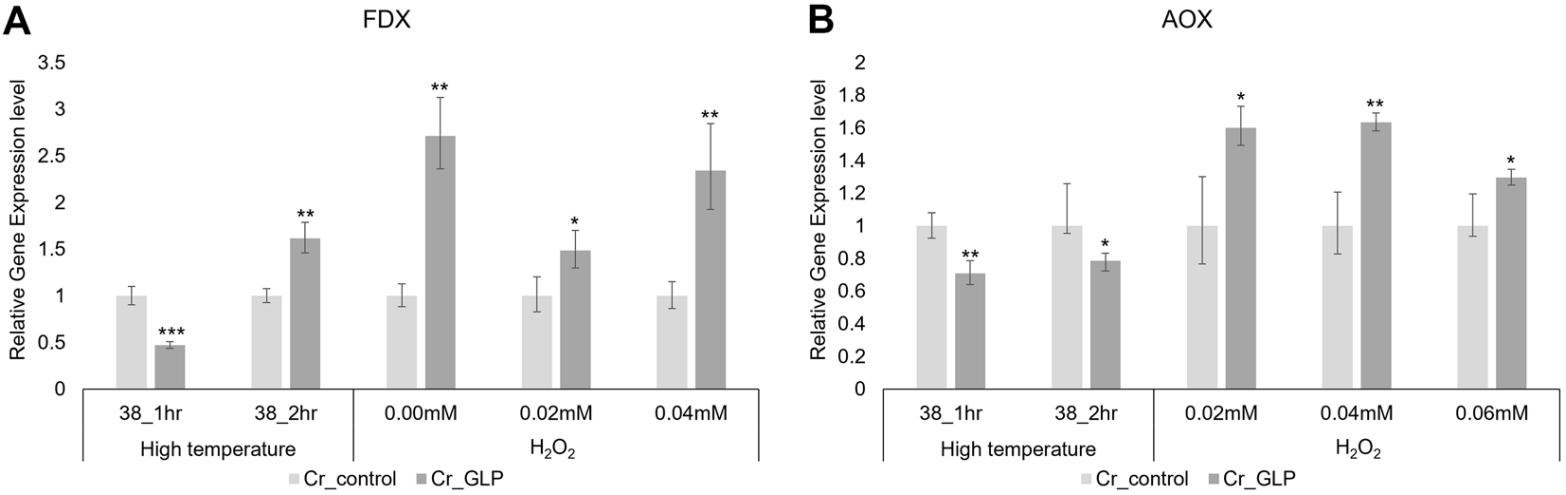
Relative gene expression levels of FDX (A) and AOX (B) of *Chlamydomonas* strains under heat and H_2_O_2_ stresses conditions, respectively.

In addition, GLP possesses oxalate oxidase activities; thus, ferredoxin (FDX), catalase (CAT), and ascorbate peroxidase (APX) related to ROS were also examined. FDX, CAT, APX gene were related with ROS-scavenging pathway (Fig. 8A) The expressions of APX and CAT, which relate to the ROS-scavenging pathway, are shown in Fig. 8. APX might be responsible for the fine modulation of ROS signaling, whereas CAT might be responsible for removing excess ROS during stress [17]. CAT is more directly associated with the pathway that removes the ROS, meaning any increase in CAT expression is focused rather than a somewhat lower decrease in APX under H_2_O_2_ conditions. Ferredoxins are components of the water–water cycle and ROS-scavenging pathway, producing ascorbate and peroxiredoxin to protect the photosynthetic apparatus [18-20]. The expression of FDX by Cr_GLP was 1.48-times, which equated to 2.34-times higher than the Cr_control under H_2_O_2_ conditions (Fig. 9A).

The alternative oxidase (AOX) is a non-energy-conserving terminal oxidase found in all plants. It bypasses proton-pumping complexes III and IV in the cytochrome pathway to transfer electrons directly from reduced ubiquinone to molecular oxygen [21]. In a previous study, the AOX gene is enhanced by abiotic and biotic stress conditions, especially temperature, such as cold stress [22]. Furthermore, some studies suggested that overexpressed AOX can enhance growth tolerance under high-temperature stress [22]. Thus, the relative expressions of AOX in Cr_GLP are 1.6 times, 1.6 times, and 1.4 times higher than Cr_control, respectively (Fig. 9B). Under saline stress, no difference was found between Cr_control and Cr_GLP in the expression of these genes.

## Discussion

This study aimed to characterize the function of the GLP gene in *C. reinhardtii* under various stress conditions. Recombinant *C. reinhardtii* expressing the GLP gene exhibited increased lipid accumulation and enhanced survival rates compared to wild-type when subjected to abiotic stress. In our previous study, putative GLP gene was identified during the comparative transcriptome analysis of N. yesoenesis (data not shown). The function of the gene was not reported yet, then we selected the specific GLP gene *N. yezoensis* in this study.

Quantitative real-time PCR was conducted to quantify the related genes with resistance to biotic and abiotic stresses. When the Cr_control and Cr_GLP were incubated under nitrogen starvation, the accumulated starch in the cell was degraded by SP, while increased GPDH and PFOR levels enhanced lipid accumulation. The acclimation process of the cell adapting to the environment through carbon partitioning has also been reported in salinity conditions [9].

The heat shock factor is an inactive monomer in the nucleus and cytoplasm in non-stress conditions. Activation of HSF1 in the monomer form is inhibited by an interaction between the heat shock protein and chaperones, such as TRiC/CCT. When protein toxicity stress such as heat shock occurs, these chaperones are released to play the role of protein folding, alongside suppressing the entry of HSF1 into the cytoplasm. Through this action, HSF1 is trimerized and accumulated in the nucleus, stimulating target gene transcriptions. When a heat shock occurs, HSPs react quickly and are expressed within 30 min to 1 h to protect the cell [15].

The ascorbate scavenging pathway can remove excessive ROS and prevent land cell death, which the positive ferredoxin of this scavenger can control. *PETF*, an FDX gene, can increase the level of ascorbate reduced by growth conditions and reduce H_2_O_2_ levels [23]. Another study demonstrated that *Chlamydomonas* overexpressing the *PETF* gene increases FDX expression levels under heat stress conditions, resulting in a strain tolerant to high temperatures [24]. Changes in the expression of CAT and APX have been observed depending on the concentration of H_2_O_2_ in the medium in mitochondrial complex III-impaired-mutants that induce less PCD within H_2_O_2_ conditions [25]. Niemeyer *et al*. demonstrated that H_2_O_2_ levels in the nucleus of *C. reinhardtii* increased after heat stress by checking the H_2_O_2_ level using hypersensitive sensors in different compartments [26].

Furthermore, oxidative stress can increase the abundance of AOX and alternative pathway respiration in *C. reinhardtii*. These two pathways regulate AOX1 transcription in response to oxidative stress [27]. A previous study suggested that the AOX transcripts and protein abundance of *C. reinhardtii* sharply increased under heat stress [28].

In this study, we characterized GLP in *C. reinhardtii*, which is the model organism for the eukaryotic system. It is not a high-temperature resistant gene obtained through artificial mutation but a gene obtained from a natural species. Although it has not been revealed what role GLP plays, the reactions under various stress conditions were examined by focusing on ROS reduction, which is an estimated property of GLP. We found that GLP is resistant to hydrogen peroxide through the quantitative expression of CAT, FDX, APX, and AOX and heat stress resistance using the quantitative expression of HSF1. Through this study, it is possible to provide insights related to GLP and paroxysm, which can provide a basis for developing a variety of species resistant to multiple abiotic stresses.

## Figures and Tables

**Table 1:** List and sequences of primers used in plasmid construction and qRT-PCR.

Gene	Sequence (5'–3')		Tm (°C)	Description
GLP	F	GCTGTGACTGCTGGTACTGT	61.0	GLP
	R	CGCGGACGAGAGAATAGCAA	61.3	
SP1	F	TCTACTTCCTGCCCGACTACAAC	63.1	Starch Phosphorylase
	R	GAACACGAACACCTCCTCCAC	63.9	
SP2	F	TCTACTTCCTGCCCGACTACAAC	63.4	Starch Phosphorylase
	R	GAACACGAACACCTCCTCCAC	58.2	
GPDH	F	AACACGCTGCACGAAAACAC	60.0	Glycerol 3-phosphate dehydrogenase
	R	TTGCTGACGCAGATGATGG	59.7	
PFOR	F	CGTGGCGGTGTTTGAGA	60.9	Pyruvate–ferredoxin oxidoreductase
	R	GGTGTTGCTGGCGATGA	60.6	
FDX	F	CGGGCAAGACGAAGACTATGG	63.1	Ferredoxin
	R	GGTAGGCGGAGCACATGAG	63.4	
HSF1	F	AACATCGTCTCATGGGGTGC	62.7	Heat shock factor 1
	R	TCCATAGGTGTTGAGCTGGC	62.1	
CAT2	F	TCAGCAACCAGTACTTCAAGGTG	61.5	Catalase
	R	ATCTCAGCGTCCCACTTGATCG	63.9	
APX2	F	AGCCCTGGAACAACACAAAGGAC	65.0	Ascorbate peroxidase
	R	AGAAGTCGCGGAAGAACAGATCC	63.7	
AOX1	F	AGAGGTGATTCGTGTGCCTG	62.2	Alternative oxidase
	R	CCAGGCACACAGATCCAAGT	62.7	
Cr_Actin	F	TGTGCATACGTGGATAGCTTG	58.2	Actin
	R	ATGACCCGCTCCTCATATCTT	59.9	
Tub A	F	CTCGCTTCGCTTTGACGGTG	62.5	Tubulin
	R	CGGCTCTTCCGCATGGTGC	63.8	
